# Effect of Ondansetron on Postoperative Pain and Vomiting after Acute Appendicitis Surgery: A Placebo-Controlled Double-Blinded Randomized Clinical Trial

**DOI:** 10.1155/2024/6429874

**Published:** 2024-06-12

**Authors:** Moein Khoori, Peyman Mirghaderi, Alireza Azarboo, Forough Jamil, Nasim Eshraghi, Alireza Abdollahzadeh Baghaei

**Affiliations:** ^1^Department of Orthopedic Surgery, Joint Reconstruction Research Center, Tehran University of Medical Sciences, Tehran, Iran; ^2^Surgical Research Society (SRS), Students' Scientific Research Center, Tehran University of Medical Sciences, Tehran, Iran; ^3^School of Medicine, Tehran University of Medical Sciences, Tehran, Iran; ^4^Department of Radiology, Faculty of Medicine, Hormozgan University of Medical Sciences, Bandar Abbas, Iran; ^5^Vali-E-Asr Reproductive Health Research Center, Family Health Research Institute, Tehran University of Medical Sciences, Tehran, Iran; ^6^Anesthesiology, Critical Care and Pain Management Research Center, Hormozgan University of Medical Sciences, Bandar Abbas, Iran

## Abstract

**Background:**

Common postoperative complications following surgery, particularly acute appendicitis surgery, include postoperative pain and vomiting, which can cause discomfort and delay recovery time.

**Methods:**

A randomized double-blinded placebo-controlled clinical trial was conducted with 80 cases of acute appendicitis of American Society of Anesthesiologists (ASA) physical status I or II and aged 18–60 y/o scheduled for appendectomy under general anesthesia. Patients were randomly divided into two equal groups: group A received 4 mg of ondansetron IV (2 ml) and group B received 2 ml of normal slain IV (placebo). Pain according to VAS, nausea and vomiting according to clinical symptoms, shivering and sedation according to the Bedside Shivering Assessment Scale (BSAS), and the Ramsay Sedation Scale (RSS) at 2, 6, 12, and 24 hours after surgery were evaluated and compared between the groups.

**Results:**

There was a significant decline in the severity of pain only at 2 hours after surgery between the ondansetron and control groups (5.3 ± 1.0 vs. 6.0 ± 1.0; *p*=0.01), not showing a difference between the groups at 6, 12, and 24 hours after appendectomy. Postoperative nausea and vomiting at 2 (5% vs. 25%; *p*=0.03) and 6 (7.5% vs. 27.5%; *p*=0.04) hours after appendectomy in the ondansetron group. At different times, the ondansetron and control groups did not differ in terms of pethidine consumption or sedation.

**Conclusions:**

In conclusion, our study found that ondansetron was effective in reducing postoperative vomiting after acute appendicitis surgery. However, it did not show a clinically significant effect on postoperative pain. This trial is registered with IRCT20230722058883N1.

## 1. Introduction

Proper postoperative pain management is one of the most crucial objectives and responsibilities of surgeons and has yet to be resolved [1]. One of the frequent pains that patients typically report is that of abdominal surgery, especially appendectomy, for which moderate to severe pain following surgery is observed [2]. With a lifetime risk of 8.6% for men and 6.7% for women, acute appendicitis is a common disease that most frequently affects people between the ages of 10 and 20 [3]. Patients perceive pain at the incision site as their most critical concern following surgery [4]. Morphine and some other drug combinations are some of the most effective pain relievers after surgery. To lessen postoperative pain, many techniques and medications are employed. Systemic opioids alone cannot adequately relieve postoperative pain and can cause undesirable side effects such as nausea, vomiting, constipation, itching, and cardiac and respiratory depression [5]. Thus, finding alternative medications with fewer side effects is crucial to controlling pain after surgery.

One of the medications that has lately come to light for pain management is ondansetron [6, 7]. Ondansetron (OND), a specific 5-HT antagonist, blocks Na channels and opioid receptors [8] in brain neurons, as shown in animal studies [9]. Additionally, when used as a local anesthetic, OND is 15 times more effective than lidocaine at producing numbness and is a good substitute [9]. Several randomized controlled trials (RCTs) suggest an overall better result for OND compared with opioids and other medications [10–12]. In a related meta-analysis, Pei et al. investigated the effect of ondansetron on the prevention of propofol injection pain, as they reported that ondansetron has a similar impact to lidocaine and magnesium sulfate to effectively reduce the pain [7].

Although thoracic and upper abdominal operations are widely acknowledged to be associated with increased levels of pain following surgery [13], the following reasons support the choice of appendectomy as the focus of the investigation: first off, surgical damage, tissue inflammation, and stimulation of the visceral nerve are some of the multifactorial causes of postappendectomy pain [14, 15]. However, the relative contribution of each factor to the overall pain experience remains poorly delineated. Secondly, despite the routine nature of appendectomy, there exists a paucity of high-quality evidence regarding the optimal management of postoperative pain, especially RCTs investigating novel treatments such as the very ondansetron, in patients undergoing this procedure. Lastly, acute appendicitis represents a prevalent surgical condition, affecting individuals across diverse demographics and age groups. Its exorbitant prevalence underscores the clinical relevance and public health significance of exploring strategies to optimize postoperative outcomes in this patient population [16]. Therefore, we aimed to investigate the effect of OND on the management of pain and nausea following the highly prevalent procedure in our center, appendectomy.

## 2. Materials and Methods

### 2.1. Ethical Statement

The national research committees have approved this trial, and all procedures were carried out in compliance with their guidelines. The study protocol was registered at the Iranian Registry of Clinical Trials (IRCT) under the registration number∗IRCT20230722058883N1∗. The study intervention and objectives were explained to the participants prior to the trial's start, and their written informed consent was then obtained.

### 2.2. Trial Design

We conducted a pragmatic, single-center, randomized, double-blinded, placebo-controlled trial in∗Shahid Mohammadi Hospital, Bandar Abbas, Iran, from 2014 to 2015∗. The trial investigators were blinded as the study was overseen by an independent data and safety monitoring board, which also looked over the intermediate analysis results. Consolidated Standards of Reporting Trials (CONSORT) guidelines were followed in this study [17]. The presence of any contraindications for using ondansetron, such as the patient's reluctance, use of any type of analgesia not defined in the hospital protocol within 24 hours before the operation (with the exception of acetaminophen (paracetamol) as a common pain management regimen, within the span of emergency room admission until surgery, with the dose varying from 500 mg to 1000 mg given every 4 to 6 hours as needed), a history of allergy to ondansetron and similar compounds, a history of drug abuse, or alcohol dependence, disorders such as cardio-respiratory, liver problems, and a history of neurological or neuromuscular or psychiatric diseases (especially a history of seizures or epilepsy), a history of suffering from chronic pain syndromes, and patients with complicated surgeries were the criteria to exclude patients from the study.

**Figure 1:**
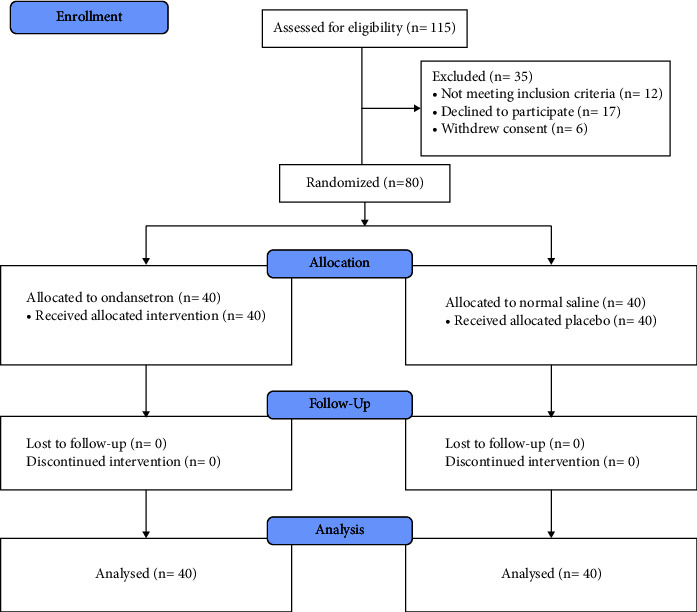
CONSORT flow diagram.

Of the 115 patients enrolled in this study, 80 patients diagnosed with appendicitis with ASA I and II, aged between 18 and 60 years, who were candidates for appendectomy, were included in the study after a full explanation of the procedure and obtaining consent (Figure 1). Shivering and sedation were measured and recorded based on the Bedside Shivering Assessment Scale (BSAS) [18] and the Ramsay Sedation Scale (RSS) [19], respectively. If a specific drug was needed to eliminate side effects, the name of the drug was written. Postoperative pain was treated with pethidine (25 mg intravenous), and shivering was treated with pethidine (25 mg intravenous) if needed (grade 4). After transferring the patients to the ward, in cases of nausea or vomiting, antiemetic drugs such as metoclopramide were used according to the patient's request. All patients underwent appendectomy under the same setting and surgical technique, with the same predetermined surgical team and the same preoperative protocol.

### 2.3. Randomization and Trial Procedures

The eligible patients were allocated at random (1 : 1) and divided into two groups by the table provided by the Random Allocation software. Group A: injection of 4 mg (2 cc) of ondansetron (drug group); group B: injection of 2 cc of normal saline (placebo group). A dedicated, password-protected web-based system was used to centrally randomize data. Patients as well as surgeons were kept unaware of the randomization until the conclusion of a 24-hour follow-up. The anesthesiologist, the patient, and the nurse in the recovery department were not informed of the content of the injected medicine.

### 2.4. Outcome Measures

At 2, 6, 12, and 24 hours, pain intensity was evaluated according to the visual analog scale (VAS) as the primary outcome. The presence or absence of nausea as well as the number of times of vomiting, the presence or absence of shivering, and sedation were secondary outcomes. To assess the clinical applicability of the differences in outcomes between cases and controls, a minimum clinically important difference (MCID) [20] was utilized.

### 2.5. Sample Size

According to the study of Zhong et al. [21], the sample size for the ondansetron group and the control group was calculated. Considering *α* = 0.05 and *β* − 1 = 0.8 and z_(1 − *α*/2) = 1.96 and z_(1 − *β*) = 1.28, the minimum sample size in each group is 38. Due to the probability of dropouts within the groups, 40 individuals were taken into consideration for each group.

### 2.6. Statistical Analysis

After collecting the required samples and information, the data were analyzed by SPSS software (version 16.0, IBM Corp.). The normality of the data was assessed using the Shapiro–Wilk test. The data exhibited an approximately normal distribution, as indicated by nonsignificant results in the Shapiro–Wilk test (*p* > 0.05). Hence, the Student's *t*-test was used to compare the two groups. Fisher's exact or the chi-square test was used to assess the categorical variables, and a *p* value of less than 0.05 was regarded as statistically significant.

## 3. Results

### 3.1. Preoperative Patient Characteristics

A total of 80 patients were included in this study, and the mean ± SD age of the total population was 27.6 ± 9.1 years, particularly 25.8 ± 8.5 in the ondansetron group and 29.4 ± 9.5 in the control group (*p*=0.1). The mean preoperative systolic and diastolic pressure, heart rate, and percentage of arterial oxygen saturation, as well as other baseline characteristics, can be observed in (Table 1). The mean VAS pain of all patients before surgery was 4.1 ± 0.9, which was 4.2 ± 0.8 in the ondansetron group and 4.1 ± 1.0 in the control group (*p*=0.8). Preoperative baseline pain and demographics were similar between the groups (all *p* > 0.05).

Regarding pain intensity, there was a significant difference between the two groups at 2 hours after the surgery (5.3 ± 1.0 in OND vs. 6.0 ± 1.0 in controls, *p*=0.01). According to the MCID, this difference was not clinically meaningful when interpreted in the context of VAS pain, as the MCID of VAS pain is reported to be a reduction of 1.37 cm in studies [22]. Other time points did not reveal significant differences between the groups (Table 2).

The mean consumption of pethidine did not differ between the OND and control groups at 2, 6, 12, and 24 hours after the operation (all *p* > 0.05). In comparison to the control group, the ondansetron group had significantly decreased rates of nausea and vomiting after 2 hours at 5% in ONDs vs. 25% in controls (*p*=0.03) and 6 hours at 7.5% in cases vs. 27.5% in controls (*p*=0.04) following surgery. The comparison of shivering and sedation variables between the two ondansetron and control groups was the same in all hours, and no difference was observed among any of the patients, and none of the patients reported shivering in the mentioned hours. Also, in the investigation of the effect of sedation, all the patients were completely calm and conscious in all the examined hours according to the Ramsay Hunt criteria (Table 2).

## 4. Discussion

Our findings suggest that ondansetron administration was associated with reduced postoperative pain at 2 hours (*p*=0.01) and a lower incidence of nausea and vomiting at 2 (*p*=0.03) and 6 (*p*=0.04) hours after surgery compared to the control group. Additionally, there were no differences in pethidine consumption or sedation between the ondansetron and control groups at various time points.

Postoperative pain is a common experience for the majority of patients who undergo surgical procedures [23], especially after acute appendicitis surgery due to tissue inflammation, nerve irritation, and surgical trauma associated with the excision of the inflamed appendix [14, 15]. Pei et al. [7] evaluated ondansetron's potential for preventing propofol injection pain, pooling a total of 782 patients from 10 RCTs. The meta-analysis revealed that the ondansetron group was associated with a decreasing incidence of propofol injection pain when compared to the control group, and this relationship was statistically significant (RR[95% CI] = 0.41[0.34, 0.49], *p* < 0.00001); there was no difference when compared to the incidence of propofol injection pain in the lidocaine group (RR[95% CI] = 1.28[0.85, 1.93], *p*=0.25); no significant differences in the incidence of propofol injection pain were observed between the ondansetron and magnesium sulfate groups (RR [95% CI] = 1.20[0.87, 1.66], *p*=0.27); when compared to the control group, the ondansetron group had a lower incidence of igniting moderate pain (RR[95% CI] = 0.37[0.26, 0.52], *p* < 0.00001) and severe pain (RR[95% CI] = 0.27[0.17, 0.43], *p* < 0.00001) during the propofol injection; however, there was no difference in the incidence of mild propofol injection pain (RR [95% CI] = 0.83[0.63, 1.10], *p*=0.20). Lee et al. [24] found no significant difference in pain levels after arthroscopic rotator cuff repair among groups in any of the time periods; except for OND (3.5 ± 1.9) vs. ramosetron (5.3 ± 2.3) and controls (5.0 ± 2.5) (*p*=0.001) in 0 to 6 hours after surgery, although in disagreement with the findings of a related meta-analysis [25]. A smaller sample size and different preoperative pain management protocols may contribute to this discrepancy. Both our and Wongyingsinn studies assessed pethidine use. Both studies found no significant difference in pethidine consumption between the ondansetron and control groups at various time points.

Being a distinctive 5-HT3 antagonist, ondansetron is an antiemetic that is frequently used to prevent postoperative nausea and vomiting (PONV) with the common dosage of 4 mg [26]. Several studies aimed to evaluate the effectiveness of ondansetron vs. other drugs in relieving postoperative nausea and vomiting (PONV). Lee et al. [24] aimed to evaluate the effectiveness of ramosetron and ondansetron vs. controls in relieving postoperative nausea and vomiting (PONV) and reported that ondansetron group had a higher frequency of complete response to administered rescue antiemetics (10 mg of metoclopramide, IV) during the 6- to 24-hour postoperative period compared to the control group (74% vs. 50%; *p*=0.01). Wongyingsinn et al. [27] focused on PONV in patients undergoing laparoscopic cholecystectomy (LC) with different interventions. The overall incidences of PONV within 24 hours of surgery were 29.1% in the fluid group (Ringer's lactate solution (10 mL/kg)), 18.4% in the ondansetron group (8 mg), and 25% in the control group, with the difference of not being statistically significant (*p*=0.4), probably due to the different dosages of ondansetron than the common protocol. Post hoc analysis in the first study revealed that patients under 50 years in the ondansetron group had significantly lower incidences of PONV and postoperative nausea (PON) than those in the control and fluid groups (0% vs. 30% and 38.5%; *p*=0.01), denoting the impact of the population's age. Two other studies evaluated the effectiveness of ondansetron in reducing the occurrence of PONV against dexamethasone [28] and metoclopramide [29] in patients undergoing laparoscopic cholecystectomy. Qasemi et al. [28] revealed that in the first 24 hours after surgery, the ondansetron group was to have less vomiting than the 8 mg dexamethasone group (8.5% vs. 20.9%; *p*=0.004) but observed no difference between the groups for postoperative nausea (*p*=0.2) which can be explained by their female-dominated population and confounding factors. Conversely, Isazadehfar et al. [29] reported a markedly lower rate of nausea in ondansetron patients than 10 mg metoclopramide (3.3% vs. 30%; *p*=0.01) but found no difference in vomiting (*p*=1). Additionally, they reported a higher number of patients on metoclopramide to need antiemetic medication than ondansetron (20% vs. 0%; *p*=0.02). The results of our study were in agreement with both of the aforementioned studies [28, 29], namely, that ondansetron is effective at reducing not only nausea but vomiting incidence.

Limitations of this study include first, the study had a relatively small sample size, which may limit the generalizability of the findings. Second, the study was conducted at a single center, which may limit the generalizability of the findings to other settings. Third, the follow-up period was only 24 hours, which may not be sufficient to fully assess the long-term effects of ondansetron on postoperative pain and vomiting. Fourth, the study did not evaluate other important outcomes, such as the length of hospital stay, time to return to normal activities, or patient satisfaction. Fifth, the study only evaluated a single dose of ondansetron, which may not reflect the optimal dose for all patients. Sixth, there was failure to investigate the effect of sedation and the intensity of shivering in the first minutes after surgery.

In addition, for further investigation and finding more accurate results, the following are suggested: first, a study to investigate the effect of different doses of ondansetron on the mentioned variables. Second, studies to check the effect of ondansetron on the mentioned variables in other surgeries. Third, the study should be conducted in more limited age groups. Fourth, a study should be conducted to investigate the intensity of pain in the mentioned hours after the local injection of ondansetron on the surgical wound. Fifth, according to the results of this study regarding shivering and sedation, i.e., the absence of shivering at all times mentioned in the study and the same degree of sedation at all hours in all patients of the two groups, it is suggested that the effect of ondansetron on the severity and occurrence of shivering and the degree and rating of sedation based on the Ramsay sedation scale criteria be investigated in the first minutes after surgery.

## 5. Conclusion

Our study concluded that ondansetron was useful in lowering postoperative nausea and vomiting following acute appendicitis surgery. It did not, however, demonstrate a clinically discernible impact on postoperative pain. To validate these results and investigate the possible advantages of ondansetron in the treatment of postoperative symptoms in patients following appendectomy, further studies with larger sample sizes are required.

## Figures and Tables

**Table 1:** Preoperative demographics and pain.

	OND	Control	Total	*p* value
Sample size	40	40	80	—
Age	25.8 ± 8.5	29.4 ± 9.5	27.6 ± 9.5	0.1
Gender (m : f)	31 : 9	27 : 13	58 : 22	0.3
BMI	24.2 ± 3.4	23.3 ± 2.6	23.8 ± 3.1	0.9
ASA (I : II)	35 : 5	33 : 7	68 : 12	0.5
Systolic BP	116.4 ± 8.5	117.4 ± 9.1	116.9 ± 8.9	0.6
Diastolic BP	74.5 ± 8.4	74.5 ± 6.9	74.5 ± 7.6	0.9
HR	85.7 ± 5.1	83.1 ± 5.3	84.4 ± 5.4	0.06
O_2_ saturation	99.4 ± 0.9	99.0 ± 0.2	99.7 ± 0.7	0.001
VAS pain	4.2 ± 0.8	4.1 ± 1.0	4.0 ± 1.9	0.8

OND, ondansetron; BMI, body mass index; m : f, male-to-female; ASA, American Society of Anesthesiologists, BP, blood pressure; HR, heart rate; VAS, visual analog scale.

**Table 2:** Comparison of postoperative outcomes between the groups.

		OND	Control	*p* value

VAS pain	2 hr	5.3 ± 1.0	6.0 ± 1.0	0.01
	2–6 hr	4.3 ± 1.0	4.5 ± 1.1	0.4
	6−12 hr	3.08 ± 1.24	2.80 ± 0.93	0.3
	12−24 hr	1.8 ± 1.0	1.4 ± 1.1	0.1
	*p* value	<0.001	<0.001	

Nausea and vomiting	2 hr	2 (5%)	10 (25%)	0.03
	2–6 hr	3 (7.5%)	11 (27.5)	0.04
	6−12 hr	2 (5%)	1 (2.5%)	1.000
	12−24 hr	0	0	—

Pethidine use	2 hr	1	1	—
	2–6 hr	0.4 ± 0.5	0.5 ± 0.5	0.4
	6−12 hr	0.1 ± 0.3	0.1 ± 0.3	0.7
	12−24 hr	0	0.02 ± 0.2	0.3
	*p* value	<0.001	<0.001	—

## Data Availability

The data that support the findings of this study are available from the corresponding author upon reasonable request.

## References

[1] Dahl J. L., Gordon D., Ward S., Skemp M., Wochos S., Schurr M. (2003). Institutionalizing pain management: the post-operative pain management quality improvement project. *The Journal of Pain*.

[2] Juhl G., Norholt S., tonnesen E., Hiesse-Provost O., Jensen T. S. (2006). Analgesic efficacy and safety of intravenous paracetamol (acetaminophen) administered as a 2 g starting dose following third molar surgery. *European Journal of Pain*.

[3] Krzyzak M., Mulrooney S. M. (2020). Acute appendicitis review: background, epidemiology, diagnosis, and treatment. *Cureus*.

[4] Phipps M. F., Monahan F. (2007). *Phipps’ Medical-Surgical Nursing Health & Illness Perspectives*.

[5] de Boer H. D., Detriche O., Forget P. (2017). Opioid-related side effects: postoperative ileus, urinary retention, nausea and vomiting, and shivering. A review of the literature. *Best Practice & Research Clinical Anaesthesiology*.

[6] Dodawad R., Sumalatha G., Pandarpurkar S., Jajee P. R. (2016). A comparative study of attenuation of propofol-induced pain by lignocaine, ondansetron, and ramosetron. *Indian Journal of Anaesthesia*.

[7] Pei S., Zhou C., Zhu Y., Huang B. (2017). Efficacy of ondansetron for the prevention of propofol injection pain: a meta-analysis. *Journal of Pain Research*.

[8] Roczniak W., Wróbel J., Dolczak L., Nowak P. (2013). Influence of central noradrenergic system lesion on the serotoninergic 5-HT3 receptor mediated analgesia in rats. *Advances in Clinical and Experimental Medicine*.

[9] Ye J. H., Mui W. C., Ren J., Hunt T. E., Wu W. H., Zbuzek V. K. (1997). Ondansetron exhibits the properties of a local anesthetic. *Anesthesia & Analgesia*.

[10] Faiz S. R., Rahimzadeh P., Nikoobakht N., Ghodrati M. R. (2015). Which one is more efficient on propofol 2% injection pain? Magnesium sulfate or ondansetron: a randomized clinical trial. *Advanced Biomedical Research*.

[11] Alipour M., Tabari M., Alipour M. (2014). Paracetamol, ondansetron, granisetron, magnesium sulfate and lidocaine and reduced propofol injection pain. *Iranian Red Crescent Medical Journal*.

[12] Zahedi H., Maleki A., Rostami G. (2012). Ondansetron pretreatment reduces pain on injection of propofol. *Acta Medica Iranica*.

[13] Saikia P., Singh P., Lahakar M. (2016). Prevalence of acute post-operative pain in patients in adult age-group undergoing inpatient abdominal surgery and correlation of intensity of pain and satisfaction with analgesic management: a cross-sectional single institute-based study. *Indian Journal of Anaesthesia*.

[14] Palabiyik O., Demir G. (2021). Chronic pain after open appendectomy and its effects on quality of life in children aged 8-18 years. *Pain Research and Management*.

[15] Kim H. O., Yoo C. H., Lee S. R. (2012). Pain after laparoscopic appendectomy: a comparison of transumbilical single-port and conventional laparoscopic surgery. *Journal of the Korean Surgical Society*.

[16] Ferris M., Quan S., Kaplan B. S. (2017). The global incidence of appendicitis: a systematic review of population-based studies. *Annals of Surgery*.

[17] Schulz K. F., Altman D. G., Moher D. (2010). CONSORT 2010 Statement: updated guidelines for reporting parallel group randomised trials. *Trials*.

[18] Badjatia N., Strongilis E., Gordon E. (2008). Metabolic impact of shivering during therapeutic temperature modulation: the Bedside Shivering Assessment Scale. *Stroke*.

[19] Ramsay M. A., Savege T. M., Simpson B. R., Goodwin R. (1974). Controlled sedation with alphaxalone-alphadolone. *British Medical Journal*.

[20] Lee J. S., Hobden E., Stiell I. G., Wells G. A. (2003). Clinically important change in the visual analog scale after adequate pain control. *Academic Emergency Medicine*.

[21] Zhong B. (2009). How to calculate sample size in randomized controlled trial?. *Journal of Thoracic Disease*.

[22] Sutton R. M., McDonald E. L., Shakked R. J., Fuchs D., Raikin S. M. (2019). Determination of minimum clinically important difference (MCID) in visual analog scale (VAS) pain and foot and ankle ability measure (FAAM) scores after hallux valgus surgery. *Foot & Ankle International*.

[23] Pirie K., Traer E., Finniss D., Myles P. S., Riedel B. (2022). Current approaches to acute postoperative pain management after major abdominal surgery: a narrative review and future directions. *British Journal of Anaesthesia*.

[24] Lee S. U., Lee H. J., Kim Y. S. (2020). The effectiveness of ramosetron and ondansetron for preventing postoperative nausea and vomiting after arthroscopic rotator cuff repair: a randomized controlled trial. *Journal of Orthopaedic Surgery and Research*.

[25] Gao C., Li B., Xu L. (2015). Efficacy and safety of ramosetron versus ondansetron for postoperative nausea and vomiting after general anesthesia: a meta-analysis of randomized clinical trials. *Drug Design, Development and Therapy*.

[26] Maharaj C. H., Kallam S. R., Malik A., Hassett P., Grady D., Laffey J. G. (2005). Preoperative intravenous fluid therapy decreases postoperative nausea and pain in high risk patients. *Anesthesia & Analgesia*.

[27] Wongyingsinn M., Peanpanich P., Charoensawan S. (2022). A randomized controlled trial comparing incidences of postoperative nausea and vomiting after laparoscopic cholecystectomy for preoperative intravenous fluid loading, ondansetron, and control groups in a regional hospital setting in a developing country. *Medicine (Baltimore)*.

[28] Qasemi F., Aini T., Ali W. (2023). The effectiveness of ondansetron and dexamethasone in preventing postoperative nausea and vomiting after laparoscopic cholecystectomy. *Cureus*.

[29] Isazadehfar K. E. M., Entezariasl M., Shahbazzadegan B., Nourani Z., Shafaee Y. (2017). The comparative study of ondansetron and metoclopramide effects in reducing nausea and vomiting after laparoscopic cholecystectomy. *Acta Medica Iranica*.

